# Short- to midterm outcomes of stemless reverse total shoulder arthroplasty: a systematic review

**DOI:** 10.1016/j.jsea.2026.100021

**Published:** 2026-04-21

**Authors:** Alisha Chan, Ajay Shah, Diane Nam, Patrick Henry, Ujash Sheth

**Affiliations:** a Sunnybrook Research Institute, Toronto, Ontario, Canada; b Sunnybrook Orthopaedic Upper Limb (SOUL), Division of Orthopaedic Surgery, Department of Surgery, Sunnybrook Health Sciences Centre, University of Toronto, Toronto, Ontario, Canada

**Keywords:** Humeral bone preservation, Reoperation, Complications, Patient-reported outcome measures, Stemless, Reverse total shoulder arthroplasty, Systematic review

## Abstract

**Background:**

Reverse total shoulder arthroplasty (rTSA) using stemless humeral components is gaining popularity. Advantages include humeral bone preservation, reduced operative time, and decreased blood loss. However, stemless implants may increase malpositioning and component loosening rates. We evaluated outcomes following stemless rTSA.

**Methods:**

Comprehensive screening of EMBASE, PubMed, and MEDLINE until June 2024 for studies reporting stemless rTSA clinical outcomes was performed according to Preferred Reporting Items for Systematic Reviews and Meta-Analyses guidelines. Included studies were assessed for methodological quality using the Methodological Index for Non-Randomized Studies (MINORS) instrument. Outcomes included demographics, functional outcomes, patient-reported outcome measures, complications, and radiographic outcomes. Stemmed and stemless implant comparisons in cohort studies were pooled for analysis.

**Results:**

Thirteen studies with moderate methodological quality were included. Five hundred ninty-two patients (598 shoulders) provided a mean follow-up of 41.8 months and age of 70.6 years. Weighted mean improvement in flexion, abduction, and Constant score were +47.8°, +42.5°, and +31.7, respectively. Complication, reoperation, scapular notching, and asymptomatic humeral component loosening rates were 14.3%, 7.0%, 17.7%, and 3.3%, respectively. Among stemless and stemmed rTSA comparative studies, no differences in patient-reported outcome measures, functional outcomes, and complications were noted.

**Conclusion:**

Stemless rTSA improved clinical outcomes, with similar complications and reoperation rates to stemmed designs. Further comparative and registry-based studies should evaluate long-term stemless rTSA outcomes.

Reverse total shoulder arthroplasty (rTSA) is the gold standard treatment for rotator cuff arthropathy.^17^ The concept of a reverse shoulder arthroplasty was first introduced by Charles Neer in 1974. Paul Grammont further developed the idea and brought forward the traditional component design, including a stemmed humeral implant.^10^^,^^31^ Variations of this design have yielded excellent outcomes over the last 20 years.^6^

As surgical indications have expanded to include younger patients who may eventually require revision surgery, the goal of preserving bone stock has become even more imperative. As such, surgeons have aimed to use short-stem and stemless protheses. The stemless design was first introduced in 2004 (Biomet Total Evolutive Shoulder System [TESS]), offering stemless humeral components for both anatomic and reverse implants.^7^

The stemless design comes with advantages such as bone stock preservation, increased reconstruction flexibility, reduced likelihood of tuberosity fractures, decreased blood loss, and decreased lateralization.^10^^,^^28^ A stemless implant also decreases the incidence of stress shielding seen in stemmed implants.^2^ However, it is not without its share of limitations. Stemless implants require robust cancellous bone and thus may limit the number of patients appropriate for this implant.^30^ Stemless implants are more prone to overloading especially in those engaging in heavy manual labor or those with larger arms.^26^ Stemmed components allow for intramedullary reference and better accuracy of the humeral osteotomy; therefore, the lack of a stem may lead to higher rates of malpositioning.^26^

Although systematic reviews have been published on this topic in the past,^2^^,^^15^^,^^19^ there has been a significant growth in the literature on stemless rTSA over the last few years. In addition, prior reviews have also included implants with short metaphyseal stems rather than evaluating the outcomes of stemless rTSA exclusively. The goal of this systematic review was to evaluate the outcomes following stemless rTSA.

## Methods

### Search strategy

Three electronic databases (EMBASE, PubMed, and Ovid [MEDLINE]) were searched from database inception until June 14, 2024, reporting on the clinical outcomes following stemless rTSA. The search included key terms: shoulder, arthroplasty, replacement, stemless (see Appendix for full search strategy).

### Study eligibility assessment

The research question and study eligibility criteria were established *a priori*. The inclusion criteria were English-language studies published in peer-reviewed journals investigating humans, patients who have undergone stemless rTSA, those reporting clinical outcomes and complications after stemless rTSA, and studies with level of evidence I to IV. Where possible, cohort studies directly comparing stemless and stemmed rTSA performance were included. Exclusion criteria were animal studies, commentaries, expert opinions, systematic reviews, book chapters, review articles, cadaver studies, biomechanical studies, and technical studies.

### Study screening

The Preferred Reporting Items for Systematic Reviews and Meta-Analyses guidelines were followed to screen studies.^18^ Titles, abstracts, and full-text articles were screened by 2 reviewers (AS, AC) independently and in duplicate. Disagreements during title and abstract screening moved onto the next stage for a more in-depth review. Any disagreements were discussed between reviewers, and a senior author was consulted for remaining discrepancies. The references of included studies were subsequently manually screened for additional articles that may have been excluded during the initial search.

### Data abstraction

Data were collected by 2 independent reviewers and recorded in a Microsoft Excel spreadsheet (version 2023, Microsoft, Redmond, WA, USA). Basic study characteristics abstracted included authors, title, country, year of publication, journal title, and study design. Patient demographics abstracted included sample sizes, sex ratio, and mean age. Patient outcomes abstracted included mean follow-up, implant type, patient-reported outcome measure (PROM) scores, and functional scores (eg, range of motion [ROM]). Complications assessed included indications for reoperations, complications that did not require reoperation, and radiographic outcomes like scapular notching, stress shielding, and implant loosening.

### Quality assessment

The methodological quality of the included studies was assessed using the MINORS instrument.^24^ It was designed to assess the methodological quality of comparative and noncomparative, nonrandomized surgical studies. In order to evaluate publication bias, hand-searching of conference abstracts from major upper-extremity meetings was carried out, and authors of unpublished data were contacted via e-mail when possible.

### Assessment of agreement

In order to assess inter-reviewer agreement, a kappa (κ) statistic was calculated for the title and abstract, and full-text screening stages. An intraclass correlation coefficient (ICC) was calculated for the quality assessment using the MINORS criteria. Agreement was categorized *a priori* as follows: κ/ICC of 0.61 or greater was considered substantial agreement; κ/ICC of 0.21 to 0.60, moderate agreement; and κ/ICC of 0.20 or less indicating slight agreement.^16^

### Statistical analysis

Given the heterogeneous nature of the studies included in this systematic review in terms of techniques and outcome reporting, some results were presented in a qualitative fashion. Descriptive statistics including weighted means, proportions, unpaired *t*-tests, chi-square tests, standard deviations, and 95% confidence intervals (CIs) were calculated using R (Version 5.4.2). When possible, quantitative outcomes were pooled as weighted means, but between-group statistical analysis could not be performed due to a lack of baseline cohort equivalence. Studies with missing data were excluded in the data analysis.

## Results

### Search strategy

**Figure 1:**
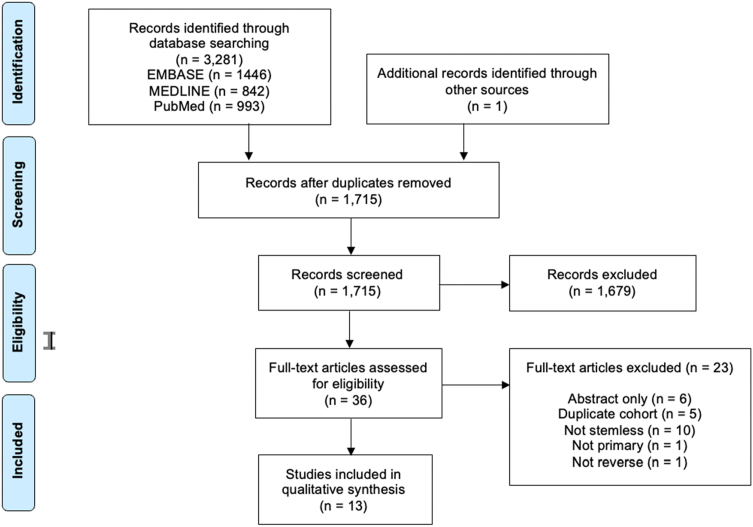
PRISMA flow diagram demonstrating the systematic review of the literature for stemless reverse total shoulder arthroplasties. *PRISMA*, Preferred Reporting Items for Systematic Reviews and Meta-Analyses.

An initial search of the databases resulted in 3,281 studies. A systematic screening and assessment of eligibility identified 13 full-text articles that satisfied the inclusion and exclusion criteria (Fig. 1). The reviewers reached substantial agreement at the title and abstract stage (κ = 0.707; 95% CI 0.596 to 0.819) and the full-text screening stage (κ = 1.00).

### Study characteristics

In total, 592 patients and 598 shoulders were included that had undergone stemless rTSA. Mean age was 70.6 years (range: 43-89, n = 598), with the weighted mean age being 70.0 years. Mean follow-up time was 41.8 months (15-142 months, n = 468). The overall male to female ratio was 229:257 (Table I).

**Table I:** Study characteristics.

Author	Journal	Yr	Country	Study design	LOE	Implant used	Patients, n (shoulders)	Mean follow-up, mo	Follow-up range, mo	Mean age, yr	Age range, yr	Gender, male/female
Ballas and Béguin^4^	J Shoulder Elbow Surg	2013	France	Retrospective case series	IV	TESS	56 (56)	59	38-95	74	55-85	16/40
Kadum et al^13^	Int Orthop	2014	Sweden	Comparative	III	TESS	16 (16)	35	15-66	69	62-76	10/6
Teissier et al^27^	J Shoulder Elbow Surg	2015	France	Retrospective case series	IV	TESS	87 (91)	41	24-69	73	55-89	61/26
von Engelhardt et al^9^	Arch Orthop Trauma Surg	2015	Germany	Retrospective case series	IV	TESS	56 (56)	17.5	NR	NS	NR	NS
Moroder et al^20^	Int Orthop	2016	Austria	Comparative	III	TESS	24 (24)	34.2	24-75	75.6	NR	7/17
Beck et al^5^	Arch Orthop Trauma Surg	2019	Germany	Retrospective case series	IV	TESS	11 (12)	101.6	75-142	72.4	53-88	5/22
Schoch et al^23^	JSES Int	2021	Germany	Retrospective case series	IV	SMR	52 (52)	29.3	24-47	61.2	46-76	NR
Galhoum et al^12^	J Shoulder Elbow Arthroplast	2022	UK	Comparative	III	Comprehensive Nano Stemless	15 (15)	27	21-33	70	63-77	NS
Nabergoj et al^21^	J Shoulder Elbow Surg	2023	France	Retrospective case series	IV	Easytech Stemless	115 (115)	NR	NR	68.7	43-88	54/61
A'Court et al^1^	J Shoulder Elbow Surg	2024	New Zealand	Retrospective cohort study	III	SMR	30 (30)	37.5	NR	64.3	NR	12/18
Antoni et al^3^	Semin Arthroplasty	2024	France	Retrospective cohort study	III	Easytech Stemless	90 (90)	31.2	NR	68.5	NR	41/49
Rosso et al^22^	J Shoulder Elbow Surg	2024	Switzerland	Retrospective case series	IV	SMR	25 (26)	46.8	25-66	70.1	59.9-86.4	NS
Willems et al^29^	JSES Int	2024	Netherlands	Retrospective case series	IV	SMR	15 (15)	NR	NR	78.3	68-86	9/6

*LOE*, level of evidence; *NR*, not reported; *NS*, not stratified; *J Shoulder Elbow Surg*, Journal of Shoulder and Elbow Surgery; *Int Orthop*, International Orthopedics; *Arch Orthop Trauma Surg*, Archives of Orthopedic and Trauma Surgery; *JSES Int* JSES International; *J Shoulder Elb Arthroplast*, Journal of Shoulder and Elbow Arthroplasty; *Semin Arthroplasty*; Seminars in Arthroplasty; *TESS*, Total Evolutive Shoulder System; *SMR*, Shoulder Modular Replacement.

### Study quality

Of the 13 included studies, all were level III or IV evidence. The mean MINORS score of the noncomparative studies was 10 (ranging 6 to 13) out of 16, corresponding to moderate quality (2 low studies <8, 5 moderate, 1 high >12), and the mean MINORS score for the comparative studies was 18.4 (ranging 16 to 20) out of 24, corresponding to moderate quality.^24^

### Surgical indication

The most common indications for surgery were rotator cuff tear/arthropathy (229 patients, 61.1%), primary osteoarthritis (53 patients, 14.1%), massive rotator cuff tear (21 patients, 5.6%) fracture sequelae (9 patients, 2.4%), and rheumatoid arthritis (9 patients, 2.4%). Three studies reported on the Hamada classification: Ballas and Béguin^4^ reported 46 shoulders in stage I, II, or III, and 10 shoulders in stage IV or V. Teissier et al^27^ recorded 45% of patients as stage III and 46% as stage IV, while Moroder et al^20^ noted 5 patients in stage II, 8 patients in stage III, 7 patients in stage IV, and 4 patients in stage V.

### Patient-reported outcome measures

**Figure 2:**
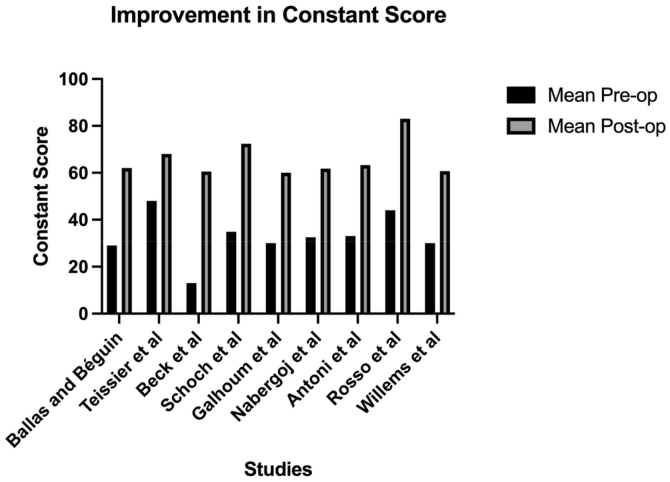
Graph demonstrating the mean improvement in the Constant score in included studies.

Studies showed consistent improvement in PROMs following stemless rTSA. The most used outcome measure was the Constant score (n = 10).^3^, ^4^, ^5^^,^^11^^,^^20^, ^21^, ^22^, ^23^^,^^27^^,^^29^ The weighted mean difference in the Constant score was +31.7 (pre-operative: 33.9, post-operative: 65.6, range: +20.0 to +47.5, n = 472) (Fig. 2).

The second-most used outcome measure was the American Shoulder and Elbow Surgeons (ASES) Shoulder score (n = 8).^1^^,^^3^^,^^11^^,^^20^, ^21^, ^22^^,^^27^^,^^29^ The weighted mean difference of the ASES Shoulder score was +40.7 (pre-operative: 38.1, post-operative: 65.9, range: +30 to +48, n = 171). Pain was measured using the visual analog scale in 7 studies,^1^^,^^5^^,^^11^^,^^13^^,^^20^^,^^22^^,^^27^ providing a weighted mean difference of −5.3 (pre-operative: 6.9, post-operative: 1.5, range: −6.8 to −2, n = 160) (Table II).

**Table II:** Patient-reported outcomes.

Author	Constant score	ASES score	QuickDASH	Oxford shoulder score	Subjective shoulder value	Visual analog scale	Additional PROMs
Ballas and Béguin^4^	Pre: 29 Post: 62 Improve: +33	NR	NR	Pre: 46/60 Post: 17/60 Improve: −29	NR	NR	NR
Kadum et al^13^	NR	NR	Pre: 67 Post: 29 Improve: −38	NR	NR	Pre: 3 Post: 1 Improve: −2	EQ-5D
Teissier et al^27^	Pre: 48 Post: 68 Improve: +20	NR	Pre: NR Post: 20/30 Improve: NR	NR	NR	Pre: 8 Post: 2 Improve: −6	NR
von Engelhardt et al^9^	NS	NR	NS	NR	NR	NR	NR
Moroder et al^20^	Pre: NR Post: 65.4 Improve: NR	Pre: NR Post: 76.2 Improve: NR	NR	NR	Pre: NR Post: 86.6 Improve: NR	Pre: NR Post: 0.4 Improve: NR	NR
Beck et al^5^	Pre: 13 Post: 60.5 Improve: +47.5	NR	Pre: 70.9 Post: 28.9 Improve: −42	NR	NR	Pre: 7.5 Post: 1.4 Improve: −6.1	NR
Schoch et al^23^	Pre: 34.9 Post: 72.4 Improve: +37.5	NR	NR	NR	Pre: 35 Post: 84.3 Improve: +49.3	NR	ADLEIR
Galhoum et al^12^	Pre: 30 Post: 60 Improve: +30	Pre: 28 Post: 76 Improve: +48	NR	NR	NR	Pre: 8 Post: 1.2 Improve: −6.8	SANE, ADLEIR
Nabergoj et al^21^	Pre: 32.5 Post: 61.8 Improve: +29.3	Pre: 36.9 Post: 78.8 Improve: +41.9	Pre: 58.1 Post: 24.5 Improve: −33.6	NR	Pre: 27 Post: 77.5 Improve: +50.5	NR	Satisfaction
A'Court et al^1^	NR	Pre: NR Post: 72.9 Improve: NR	NR	Pre: NR Post: 40.1/48 Improve: NR	NR	Pre: NR Post: 2 Improve: NR	Likert scale for satisfaction
Antoni et al^3^	Pre: 33 Post: 63.3 Improve: +30.3	Pre: NR Post: 78.9 Improve: NR	Pre: NR Post: 24.5 Improve: NR	NR	NR	NR	NR
Rosso et al^22^	Pre: 44 Post: 83 Improve: +39	Pre: 56 Post: 86 Improve: +30	Pre: 41 Post: 18 Improve: −23	NR	Pre: 44.3 Post: 85.3 Improve: +41	Pre: 4.6 Post: 0.9 Improve: −3.7	UCLA Shoulder Score
Willems et al^29^	Pre: 30 Post: 60.7 Improve: +30.7	Pre: 25.8 Post: 69 Improve: +43.2	NR	Pre: 18.6/48 Post: 36.6/48 Improve: +18	NR	NR	EQ-5D-5 L, satisfaction

*NR*, not reported; *NS*, not stratified; *PROMs*, patient-reported outcome measures; *ASES*, American Shoulder and Elbow Surgeons; *QuickDASH*, Quick Disabilities of the Arm, Shoulder and Hand; *ADLEIR*, activities of daily living requiring external and internal rotation; *SANE*, Single Assessment Numeric Evaluation; *UCLA*, University of California Los Angeles; *EQ-5D*, EuroQol 5 Dimension; *EQ-5D-5 L*, EuroQol 5 Dimension 5 Level.

### Functional outcomes

Ten studies^1^^,^^4^^,^^5^^,^^11^^,^^13^^,^^21^, ^22^, ^23^^,^^27^^,^^29^ reported ROM outcomes using degrees. The overall weighted mean difference in flexion was +47.8° (pre-operative: 89.0°, post-operative: 135.4°, range: +28 to +88, n = 398. The weighted mean difference in abduction was +42.5° (pre-operative: 81.8°, post-operative: 122.6°, range: +23 to +80, n = 301). External rotation was only recorded in 7 of the 13 studies using degrees, while internal rotation was recorded in 8 of the studies each with varying measurement systems, precluding pooled analysis (Table III).

**Table III:** Range of motion.

Author	Flexion (degrees)	Abduction (degrees)	External rotation (degrees)	Internal rotation
Ballas and Béguin^4^	Pre: 79 Post: 140 Improve: +61	NR	Pre: 13 Post: 45 Improve: +32	NR
Kadum et al^13^	Pre: 50 Post: 110 Improve: +60	Pre: 30 Post: 110 Improve: +80	NR	Pre: sacrum Post: L3
Teissier et al^27^	Pre: 96 Post: 143 Improve: +47	Pre: 89 Post: 138 Improve: +49	Pre: 26 Post: 39 Improve: +13	Pre: 5 (CS) Post: 4 (CS) Improve: −1
von Engelhardt et al^9^	NR	NR	NR	NR
Moroder et al^20^	Pre: NR Post: 7.8 (CS) Improve: NR	Pre: NR Post: 6.9 (CS) Improve: NR	Pre: NR Post: 6.6 (CS) Improve: NR	Pre: NR Post: 5.3 (CS) Improve: NR
Beck et al^5^	Pre: 51.9 Post: 135.5 Improve: +83.6	Pre: 38.3 Post: 116.1 Improve: +77.8	NR	NR
Schoch et al^23^	Pre: 87 Post: 138 Improve: +51	Pre: 72 Post: 130 Improve: +58	Pre: 15 Post: 28 Improve: +13	NR
Galhoum et al^12^	Pre: 82 Post: 110 Improve: +28	Pre: 77 Post: 100 Improve: +23	Pre: 15 Post: 45 Improve: +30	Pre: 28.6% at thigh, 21.4% at buttock, 28.6% at sacroiliac, 14.3% at L3, 7.1% at D9 Post: 7.1% at thigh, 35.7% at buttock, 42.9% at sacroiliac, 7.1% at L3, 7.1% at D9
Nabergoj et al^21^	Pre: 107 Post: 136 Improve: +29	Pre: 93 Post: 117 Improve: +24	NR	Pre: 43% at buttock, 34% at sacrum, 20% at L3, 3% at T7/12 Post: 22% at buttock, 20% at sacrum, 38% at L3, 21% at T7/12
A'Court et al^1^	Pre: NR Post: 117 Improve: NR	Pre: NR Post: 105 Improve: NR	Pre: NR Post: 31 Improve: NR	Pre: NR Post: Mid-lumbar Improve: NR
Antoni et al^3^	NR	NR	NR	NR
Rosso et al^22^	Pre: 66 Post: 154 Improve: +88	NR	Pre: 27 Post: 25 Improve: −2	Pre: L2 Post: L3 Improve: −1
Willems et al^29^	Pre: 71.4 Post: 115.5 Improve: +44.1	NR	Pre: 25.9 Post: 30 Improve: +4.1	Pre: 67% above sacroiliac joint Post: 92% above sacroiliac joint Improve: +25%pt

*NR*, not reported; *NS*, not stratified; *CS*, Constant score.

### Complications and reoperations

A total of 75 complications were reported in 525 stemless implants (14.3%), 37 of which required reoperation (7.0%) (Table IV). Table V notes respective rates of all complications.

**Table IV:** Complications and Reoperations.

Author	Complications, n (%)	Reoperations, n (%)
Ballas and Béguin^4^	9 (16%)	5 (9%)
Kadum et al^13^	4 (25%)	4 (25%)
Teissier et al^27^	3 (3%)	1 (1%)
von Engelhardt et al^9^	NS	NS
Moroder et al^20^	6 (25%)	2 (8%)
Beck et al^5^	NS	NS
Schoch et al^23^	3 (6%)	2 (4%)
Galhoum et al^12^	4 (27%)	4 (27%)
Nabergoj et al^21^	20 (17%)	9 (8%)
A'Court et al^1^	10 (33%)	3 (10%)
Antoni et al^3^	5 (6%)	3 (3%)
Rosso et al^22^	9 (36%)	1 (4%)
Willems et al^29^	2 (13%)	2 (13%)

*NS*, not stratified.

**Table V:** Indications and rates of overall complications.

Overall complications	Cases, n	% of complications
Glenoid component loosening	12	16.0
Periprosthetic fracture	9	12.0
Humeral (corolla) component displacement	8	10.7
Infections	6	8.0
Acromial fracture	5	6.7
Instability	3	4.0
Hematoma	3	4.0
Hand dysesthesia	3	4.0
Implant malpositioning	3	4.0
Post-operative stiffness	3	4.0
Radiographic subsidence	2	2.7
Post-operative humeral migration	2	2.7
Traumatic proximal humeral fracture	2	2.7
Unreported	2	2.7
Subscapularis rupture	1	1.3
Scapular spine stress fracture	1	1.3
Clavicle fracture	1	1.3
Biceps tenodesis tear	1	1.3
Moved implant	1	1.3
Asymmetrical polyethylene	1	1.3
Glenoid ossification	1	1.3
Cerebrovascular stroke affecting shoulder stability	1	1.3
Intraoperative partial humeral metaphyseal crack	1	1.3
Glenoid and humeral component loosening	1	1.3
Chronic scapulothoracic conflict	1	1.3
Symptomatic mesoacromion	1	1.3

The most common reason for reoperation was glenoid component loosening, comprising 32.4% of all reoperations (Table VI). Out of 37 reoperations, 17 were revisions to a stemmed component (45.9%). The remaining 38 complications without reoperations included 6 periprosthetic fractures (15.8%), 5 acromial fractures (13.2%), 3 cases of hand dysesthesia (7.9%), 3 implant malpositioning (7.9%), 3 cases post-operative stiffness (7.9%), 3 infections (7.9%), 2 humeral implant displacements (5.3%), 2 radiographic subsidence (5.3%), 1 intraoperative partial humeral metaphyseal crack (2.6%), 1 asymmetrical polyethylene (2.6%), 1 glenoid ossification (2.6%), 1 subscapularis rupture (2.6%), 1 scapular spine stress fracture (2.6%), 1 clavicle fracture (2.6%), 1 tear of the biceps tenodesis (2.6%), 1 hematoma (2.6%), and 1 moved implant (2.6%).

**Table VI:** Indications and rates of Reoperations.

Indications for reoperations	Cases, n	% of reoperations
Glenoid component loosening	12	32.4
Humeral (corolla) component displacement	6	16.2
Infections	3	8.1
Instability	3	8.1
Periprosthetic fracture	3	8.1
Post-operative humeral migration	2	5.4
Traumatic proximal humeral fracture	2	5.4
Cerebrovascular stroke affecting shoulder stability	1	2.7
Hematoma	1	2.7
Glenoid and humeral component loosening	1	2.7
Chronic scapulothoracic conflict	1	2.7
Symptomatic mesoacromion	1	2.7
Unreported	1	2.7

Out of 85 stemmed rTSA patients, there were 10 reported complications (11.8%), 8 of which required reoperation (9.4%). A comparison of the complication and reoperation rates between the stemmed rTSA and its corresponding stemless cohort were not statistically significant (*P*= .80 for complication rates, *P*= .94 for reoperation rates). Indications for reoperation included 2 hematomas (25.0%), 2 glenoid loosening (25.0%), 1 inlay snapping (12.5%), 1 aseptic baseplate loosening (12.5%), 1 greater tuberosity fracture (12.5%), and 1 acromial stress fracture (12.5%). The remaining 2 complications included 1 case of transient paresthesia and 1 case of pain over the lateral arm.

### Radiographic outcomes

Radiographic findings of scapular notching and asymptomatic humeral component loosening were reported to be rates of 17.7% and 3.3%. respectively. Moroder et al^20^ reported no loosening in either group; however, the stemmed group reported 9 cases of scapular notching (5 cases of grade 1 scapular notching, 4 cases of grade 2 scapular notching), while the stemless group noted 2 cases (2 cases of grade 1 scapular notching). Kadum et al^13^ demonstrated comparable cases of scapular notching, with 12 shoulders total from 4 patients in the stemless group and 5 patients in the stemmed group. They did not stratify their scapular notching grade results by implant type.

Six studies^3^^,^^5^^,^^9^^,^^20^^,^^21^^,^^27^ noted the humeral neck-shaft angle (NSA), providing a 147.1° weighted mean (n = 388, mean: 146.2°, median: 146.9°). Nabergoj et al^21^ observed a higher NSA in patients with scapular notching (*P*< .0008, mean of 150° with scapular notching, 142° without scapular notching). Moroder et al^20^ reported that an NSA greater than the standard 155° of the Grammont design may be correlated with a decreased notching rate in their stemless group.

### Stemless vs. stemmed reverse total shoulder arthroplasty

Three studies^1^^,^^13^^,^^20^ compared the stemless rTSA outcomes to stemmed rTSA. There was no statistical difference in baseline age between the stemmed and stemless rTSA in the 3 comparative studies (*P*= .33). Moroder et al^20^ compared 24 stemless patients to 24 stemmed patients and noted no statistically significant difference between the 2 groups in PROMs or ROM (*P*> .05 in Constant score, ASES Shoulder score, Subjective Shoulder Value, visual analog scale, and ROM) after a mean follow-up of 35 months. A'Court et al^1^ found no statistical difference between 30 stemless and 30 stemmed rTSA after a mean follow-up of 37.5 months when comparing PROMs, ROM, and post-operative complications. Kadum et al^13^ evaluated 16 stemless patients to 15 stemmed patients and found no statistically significant differences in PROMs, ROM, or complications (scapular notching, humeral loosening) at 39 months.

### Implant type

The TESS system (Biomet, Inc., Warsaw, IN, USA) was used in 6 studies,^4^^,^^5^^,^^9^^,^^13^^,^^20^^,^^27^ while the Shoulder Modular Replacement (SMR) stemless reverse implant (Lima Corporate, Villanova, San Daniele del Friuli, Italy) was used in 4 studies.^1^^,^^22^^,^^23^^,^^29^ Easytech Stemless replacements (FX Solutions, Viriat, France) were used in 2 studies,^3^^,^^21^ and Comprehensive Nano Stemless (Biomet, Inc., Warsaw, IN, USA) was used in 1 study.^11^ Pooled analysis was conducted to compare outcomes between the TESS system and the SMR implant. There was no significant difference between TESS and SMR regarding the mean improvement in Constant score (TESS: +31.24 vs. SMR: +31.81, *P*= .47). Other PROMs lacked sufficient data for pooled analysis. While the TESS implant system performed better in the mean improvement of external rotation (TESS: +19.9° vs. SMR: +7.37°, mean difference +5.50°, 95% CI + 2.23° to +8.76°, *P*< .05), SMR performed better in the mean improvement of flexion (TESS: +54.74° vs. SMR: +60.2°, mean difference +12.54°, 95% CI + 10.37° to +14.71°, *P*< .05).

The TESS and the SMR implant had comparable revision and complication rates. TESS had an 11.8% complication rate (22 of 187 shoulders), while the SMR implant had an 19.5% rate (24 of 123 shoulders) (*P*= .90). The TESS had a revision rate of 6.4% (12 of 123 shoulders), while the SMR implant had a rate of 6.5% (8 of 123 shoulders) (*P*= .95). The most common indication for revision of the TESS was for a component dislocation of either the corolla or glenoid component (n = 6). The most common indications for revision of the SMR implant was periprosthetic fractures and instability (n = 2 each).

## Discussion

This review provided an update on midterm outcomes following stemless rTSA. The most significant finding in this systematic review is that outcomes following stemless rTSA are consistently positive in PROMs, functional outcomes, and radiographic findings.

When comparing our pooled stemless data, this study shows comparable results to other reported outcomes following stemmed rTSA in the literature. The mean difference in Constant score and ASES score in this study is comparable to stemmed rTSA outcomes in a recent review of 20 studies by Doyle et al^8^ which reported mean improvement in Constant score of +34.2 and ASES of +44.5 in 1,591 shoulders at 5-year follow-up. A stemmed rTSA review by Galvin et al^12^ at a similar follow-up time to our study of 3.9 years also notes similar improvements in Constant score (+37) and ASES (+42). Functional outcomes were similar, with our study reporting improvements in forward flexion and abduction similar to that of Doyle et al^8^ (flexion +50°, abduction +42°). Galvin et al^12^ reported greater improvements of flexion and abduction of +56° and+50°, respectively.

Our review demonstrated similar rates of complications and revisions between stemless and stemmed rTSA patients. Stemless rTSA patients in our study had a 14.3% complication rate at 41.8 months, which is similar to a systematic review by Smith et al^25^ on stemmed rTSA of 14.7%. A systematic review by Zumstein et al^32^ on stemmed rTSA at a minimum of 24 months of follow-up note an overall complication rate of 24.0% (188 complications of 782 cases), higher than this study. However, Galvin et al^12^ note a much lower complication rate of 9.4%. Doyle et al^8^ does not report an overall complication rate; however, they noted the highest rate of complications overall of 4.3% due to periprosthetic infection. In addition, they reported glenoid and humeral periprosthetic fractures with an overall 3.2% rate. Our study notes an overall lower rate of periprosthetic fractures; however, it is the most common complication in our study. The overall reoperation rate in this systematic review was 7.0%. Zumstein et al^32^ showed an overall reoperation rate of 13.4%, while Galvin et al^12^ note a reoperation rate of 5.5%. Doyle et al^8^ reported a 4.9% revision rate at 106 ± 31 months, and a 14% revision rate in studies with >10-year follow-up.^25^

Scapular notching was seen in 17.7% of patients, primarily grade 1-2 changes observed. Doyle et al^8^ reported an overall rate of notching in 30.9% of shoulders, and the rate of grade 3-4 notching was 10.4%. As expected, notching was more frequent in implants with 155° NSA compared to 145° NSA implants. Thus, the complication of scapular notching may be independent of the type of humeral component implanted. This finding may also be explained by the lower mean NSA in our sample (n = 388, weighted mean: 147.1°), with few studies reporting >155° NSA. However, the relatively shorter follow-up period in the current study (41.8 months vs. 111 months) may also influence this finding. The incidence of aseptic humeral loosening in our study was 3.3%, comparable to 2.0% in the study by Doyle et al^8^ and similar to those reported by Keener et al^14^ and Zumstein et al^32^

Our study found 4 cases of greater tuberosity osteolysis; however, none were found to have impacted the humeral component.^1^^,^^4^ Another case showed resorption of the greater tuberosity after a dislocation caused a greater tuberosity fracture, which caused resorption.^21^ Twenty cases of humeral loosening were found. One case of gross humeral loosening was reported by Schoch et al^23^ win which the humeral implant was not at the required depth of implantation; however, no reoperation was done. Nabergoj et al^21^ demonstrated 5 cases of humeral loosening, 1 of which also reported glenoid loosening and required reoperation. This patient was suspected to have septic loosening through 1 positive *Cutibacterium acnes* culture out of 6. Willems et al^29^ noted 2 patients with humeral loosening found immediately post-operatively, who were revised to a stemmed implant. The remaining 12 patients were reported by Antoni et al,^3^ in which it is noted to be radiolucency or loosening under the humeral implant. No further information was given. One case of humeral component failure was reported by von Engelhardt et al,^9^ which was caused by intraoperative malpositioning. It was found on x-ray 1-2 days postsurgery and were immediately revised. Only one study was noted to have used a humeral head autograft for impaction grafting in the humerus.^23^

This study also compared the most common implant systems used, the TESS and Lima SMR. No significant differences were found in the Constant score between implants. Regarding ROM, TESS performed better in external rotation, while SMR performed better in flexion. SMR had a higher complication rate; however, both had comparable revision rates.

Patient selection bias must be taken into consideration, as stemless rTSA can only be performed in those with high quality bone stock. However, 1 study in our review compared stemless implants in patients <75 and >75 years old and found no difference in outcomes, indicating that age alone should not discriminate from implant use.

### Limitations

The primary weakness of this study is the limited number of high-quality studies included. Publication bias was addressed by searching conference abstracts for negative trials and contacting authors of unpublished data. Though authors were contacted, no unpublished data were provided and therefore could not be included in our study. The quality of included studies fell below the global ideal MINORS score (16 for noncomparative studies, 24 for comparative studies),^24^ with the included studies at 10 for noncomparative studies and 18.4 for comparative studies. The authors of 8 eligible studies received consulting fees, sponsorships, and/or funding from the companies whose implants were used, suggesting potential sponsorship bias.

The heterogeneity in outcome measures precluded a meta-analysis. Various studies only published post-operative data, utilized nonstandardized measurements, or did not adequately report data for pooled analysis. For example, von Engelhardt et al^9^ stratified their data based on the patient diagnosis before the rTSA (cuff tear arthropathy or revision arthropathy), rather than based on stemmed or stemless implant. Moroder et al^20^ reported their ROM using a Constant score, while other studies used degrees.

Moving forward, further well-designed long-term prospective studies comparing stemmed and stemless rTSA designs are needed to better understand the long-term outcomes of stemless prostheses. Registry and database studies should also be employed to evaluate the long-term survival of these implants.

## Conclusion

The current systematic review found that stemless rTSA provides comparable patient-reported and functional outcomes to stemmed rTSA at short- and midterm follow-up. Complications, revisions, and radiographic findings were also similar. However, further study is needed to evaluate long-term functional outcomes and implant survivorship.

## Disclaimers:

Funding: No funding was disclosed by the authors.

Conflicts of interest: The authors, their immediate families, and any research foundations with which they are affiliated have not received any financial payments or other benefits from any commercial entity related to the subject of this article.

## Data availability

All data generated or analyzed during this review are included in this published article.
