# Supplementary material for: Short- to midterm outcomes of stemless reverse total shoulder arthroplasty: a systematic review
Source: J Shoulder Elb Arthroplast. 2026 Apr 21;10(3):100021. doi: 10.1016/j.jsea.2026.100021 (PMC13213293; doi:10.1016/j.jsea.2026.100021)
Supplement: Supplemental Material [file mmc1.docx]

**Supplemental Material**

**Appendix Table A:** Search strategy

| EMBASE: 1446 Studies | | MEDLINE: 842 Studies | | PubMed: 993 Studies | |
| --- | --- | --- | --- | --- | --- |
| Strategy | Studies | Strategy | Studies | Strategy | Studies |
| 1. stem*.mp. | 1039828 | 1. stem*.mp. | 679872 | ((((stem* OR unstem*))) AND ((((shoulder) OR shoulder[MeSH Terms]) OR joint, shoulder[MeSH Terms]) OR joints, shoulder[MeSH Terms])) AND ((((arthroplasty) OR replacement) OR arthroplasty[MeSH Terms]) OR arthroplasty, replacement[MeSH Terms]) | 993 |
| 1. unstem*.mp. | 14 | 1. unstem*.mp. | 9 |  |  |
| 1. exp shoulder muscle/ or exp shoulder injury/ or exp shoulder/ or exp shoulder disease/ | 129587 | 1. exp SHOULDER JOINT/ or exp SHOULDER/ or shoulder.mp. or exp SHOULDER INJURIES/ | 101655 |  |  |
| 1. shoulder.mp. | 136687 | 1. arthroplasty.mp. | 115638 |  |  |
| 1. exp shoulder arthroplasty/ or arthroplasty/ or exp replacement arthroplasty/ or exp total shoulder arthroplasty/ exp total arthroplasty | 77645 | 1. ARTHROPLASTY, REPLACEMENT/ or ARTHROPLASTY/ or ARTHROPLASTY, REPLACEMENT, SHOULDER/ | 19339 |  |  |
| 1. arthroplasty.mp. | 136006 | 1. replacement.mp. | 384235 |  |  |
| 1. replacement.mp. | 530898 | 1. 1 or 2 | 679873 |  |  |
| 1. 1 or 2 | 1039830 | 1. 3 and 7 | 1614 |  |  |
| 1. 3 or 4 | 171292 | 1. 4 or 5 or 6 | 419320 |  |  |
| 1. 5 or 6 or 7 | 611862 | 1. 8 and 9 | 897 |  |  |
| 1. 8 and 9 and 10 | 1503 | 1. limit 10 to english language | 842 |  |  |
| 1. limit 11 to english language | 1446 |  |  |  |  |
